# Management of Expected Intraocular Pressure (IOP) Elevation During Prolonged Steep Trendelenburg Positioning: A Case Report and Literature Review

**DOI:** 10.7759/cureus.91017

**Published:** 2025-08-26

**Authors:** Nicole Terfloth, Jack Jerit, Claire Wright

**Affiliations:** 1 Department of Ophthalmology, The University of Tennessee Health Science Center, Memphis, USA

**Keywords:** acetazolamide, glaucoma, intraocular pressure (iop), perioperative management, primary open-angle glaucoma (poag), trendelenburg position

## Abstract

This article presents a case of intraocular pressure (IOP) assessment and perioperative management in a patient with primary open-angle glaucoma undergoing a prolonged surgical procedure in steep Trendelenburg (ST) positioning. The patient was administered prophylactic extended-release oral acetazolamide prior to an uncomplicated cystectomy to mitigate the anticipated IOP elevation associated with the surgical positioning. The patient's IOP was monitored postoperatively at a clinic visit, where it remained stable at 11 mmHg in the right eye and 9 mmHg in the left eye with preserved visual acuity and no evidence of glaucomatous progression on retinal nerve fiber layer (RNFL) and visual field testing. In addition, a review of the existing literature on perioperative IOP management in glaucoma patients undergoing ST procedures was conducted. While prior reports have explored alternative pharmacologic interventions, this case highlights the successful use of acetazolamide as a prophylactic strategy for maintaining IOP control in a primary open-angle glaucoma patient.

## Introduction

Glaucoma is a heterogeneous group of diseases characterized by progressive loss of optic nerve retinal ganglion cells and their axons, leading to structural damage and resultant visual field defects [1]. Glaucoma is the second leading cause of blindness in the United States and often progresses insidiously with peripheral vision loss as the most common presenting symptom [1]. Glaucoma is categorized as either primary or secondary and open-angle or closed-angle. Primary glaucoma develops without a known cause, while secondary glaucoma results from an underlying ocular or systemic condition. Open-angle glaucoma is defined by an anatomically open anterior chamber angle allowing aqueous humor outflow, while closed-angle glaucoma has a characteristic obstruction of this angle and reduced aqueous humor outflow [2]. 

The most common type of glaucoma is primary open-angle glaucoma (POAG), which accounts for approximately 74% of all glaucoma cases worldwide [3]. The burden of POAG is significant and increasing, affecting 2.71 million people in 2011, 4.32 million people in 2025, and projected to affect 7.32 million people by 2050 [3]. 

Elevated intraocular pressure (IOP) is a modifiable risk factor for glaucoma-induced optic neuropathy and contributes directly to optic nerve damage and retinal ganglion cell death [4]. It is well-established in the literature that increases in IOP lead to mechanical strain on the optic nerve head and retinal ganglion cells (RGC), which contribute to glaucoma-induced optic neuropathy [4]. Recent studies have shown that acute repetitive spikes in IOP can further this neuropathy by disrupting intraocular homeostasis and causing RGC death, which suggests that even transient, non-ischemic IOP spikes can further glaucoma progression [5]. There is also evidence that a single spike in IOP can lead to acute secondary optic neuropathy through reduced optic nerve head perfusion [6]. Therefore, the primary treatment strategy utilized to prevent further glaucoma-induced optic neuropathy is IOP reduction. 

IOP reduction is typically achieved through pharmaceutical therapies such as beta blockers and carbonic anhydrase inhibitors that reduce aqueous humor production or prostaglandin analogs that increase uveoscleral outflow. External factors can also affect IOP. Notably, steep Trendelenburg (ST) positioning during urogynecology procedures has been shown to cause significant, up to nearly doubled, transient increases in IOP [7]. Previous research has shown that both operative time and baseline IOP influence the degree of IOP elevation [8]. This IOP elevation has been shown to reach its maximum after 142 minutes of operative time [9]. As patients with preexisting glaucoma have compromised optic nerve health, these ST-induced intraoperative IOP spikes pose a risk of further optic neuropathy in this patient population. 

Despite the well-documented physiological impact of ST on IOP, there are no formal guidelines currently addressing perioperative IOP management in this context. In this case report, the authors describe their perioperative IOP management for a patient with POAG undergoing prolonged surgery in ST, as well as review the existing literature on proposed strategies for reducing transient IOP elevations in similar surgical settings. 

## Case presentation

A 65-year-old female patient with a history of POAG was scheduled to undergo a laparoscopic robotic cystectomy for urothelial carcinoma removal in September 2023. The procedure required the patient to be in prolonged ST for six hours. The patient had a diagnosis of severe POAG in the right eye and mild POAG in the left eye based on retinal nerve fiber layer (RNFL) analysis and visual field testing. The patient had undergone combined cataract extraction with intraocular lens (CEIOL) with Kahook Dual Blade (KDB) goniotomy (New World Medical, Cucamonga, CA) in both eyes in 2022, with IOP well-controlled off glaucoma drops. Of note, the patient had axial myopia and underwent laser-assisted in situ keratomileusis (LASIK) in both eyes in the 1990s, with post-LASIK central corneal thickness measured at 470 µm in the right eye and 469 µm in the left eye. 

Prior to her cystectomy, the patient expressed concern after reading reports in the medical literature indicating that prolonged Trendelenburg positioning can lead to significant transient increases in IOP and the potential for further optic nerve damage. She raised these concerns with her anesthesia and surgical team, who referred her to her ophthalmologist for perioperative evaluation and recommendations. Her IOP at her pre-cystectomy ophthalmic appointment was 12 mmHg in each eye via Goldmann applanation tonometry. Previous fundus photos showed marked cupping with a scleral crescent in the right eye and moderate cupping with a scleral crescent in the left eye.

Given her history of advanced glaucoma in the right eye, a prophylactic IOP-lowering regimen was proposed to minimize the risk of intraoperative optic nerve damage. The patient was prescribed oral extended-release acetazolamide and instructed to take one 500 mg tablet in the pre-operative area. The patient was also advised by her ophthalmologist to communicate with the anesthesia team prior to surgery regarding the potential need for an additional optional 500 mg IV dose of acetazolamide. 

The patient underwent an uncomplicated cystectomy with estimated blood loss measuring 200 mL and case time measuring seven hours and 12 minutes. 

At the patient’s subsequent ophthalmic clinic visit, the patient confirmed that she took one tablet of oral acetazolamide as instructed in the preoperative area without any reported side effects. No acetazolamide was administered intraoperatively by the anesthesia team. Her IOP was 11 mmHg in the right eye and 9 mmHg in the left with stable visual acuity, and there was no concern for progression on subsequent RNFL and visual field testing. Fundus photos taken six months after her cystectomy showed stable marked cupping in the right eye and stable moderate cupping in the left eye.

## Discussion

Review of the literature:

The authors identified one previously reported case of a patient with POAG who was treated perioperatively for ST-induced IOP spikes [10] and six experimental studies in the literature that tested different perioperative treatment modalities on healthy patients without ocular disease to determine potential strategies for reducing ST-induced IOP spikes [11-17]. The literature review included case reports, case series, randomized controlled trials, and systematic reviews published between January 2000 and May 2025. These cases are summarized in Table 1.  

**Table 1:** Interventional strategies in the literature aimed at reducing increases in IOP during ST positioning ST: steep Trendelenburg; RCT: randomized controlled trial; IOP: IOP: intraoperative pressure

Author	Population	Type of study	Intervention	Timing of intervention	Duration in ST position (minutes)	Effect on IOP	IOP measurement method
Joo et al., 2016 [10]	Healthy adults	RCT	1.0 µg/kg loading dose of dexmedetomidine + 0.5 µg/kg/h infusion	Loading dose prior to anesthesia + infusion during operation	120-240	No statistically significant difference between experimental and control groups	Tonopen
Kim et al., 2015 [11]	Healthy adults	RCT	0.4 µg/kg/h of dexmedetomidine	Following the induction of anesthesia	180	At 60 minutes, the experimental group had a lower IOP than the control group	Tonopen
Kitamura et al., 2018 [12]	Healthy adult males	RCT	0.4 µg/kg/h of dexmedetomidine	Following the induction of anesthesia	180	At 180 minutes, the experimental group had lower IOP than the control group	Tonopen
Lee et al., 2016 [13]	One patient with severe POAG	Case Study	500 mg IV bolus of acetazolamide + 20% mannitol infusion	When the IOP reached 35 mmHg	120	Decrease in IOP 30 minutes after bolus was initiated + maintenance of in-range IOP with infusion	Tonopen
Molloy, 2014 [14]	Healthy adults	RCT	Dorzolamide-timolol	When the IOP reached 40 mmHg	157-273	At 60, 90, and 120 minutes, the experimental group had lower IOP than the control group	Tonopen
Molloy et al., 2016 [15]	Healthy adults	RCT	Dorzolamide-timolol	Following anesthesia induction	180	The treatment group had lower IOP compared to the control group	Tonopen
Raz et al., 2015 [16]	Healthy adults	RCT	Z-Trendelenburg position	Following anesthesia induction	300	Mean decrease in IOP of 4.6 mmHg	Tonopen
Yoo et al., 2014 [17]	Healthy adults	RCT	Propofol vs. sevoflurane as an anesthetic agent	At anesthesia induction	65-126	No significant differences in the two groups at any time point	Tonopen

Head positioning, particularly when the head is positioned inferior to the feet, is known to increase IOP. Such positioning occurs in ST, as well as during certain yoga exercises such as Sirsasana (headstand pose), Halasana (plow pose), and Adho Mukha Svanasana (downward-facing dog pose) [18]. ST positioning is thought to elevate IOP through increasing resistance to aqueous humor outflow. This increased resistance leads to venous congestion and a subsequent rise in IOP. ST positioning has also been reported to affect ocular perfusion pressure (OPP), which influences other IOP regulatory factors, including choroidal and retinal circulation [19]. Patients with glaucoma, by definition, have preexisting optic neuropathy and thus have a lower threshold to develop additional optic nerve damage with even transient IOP elevations, such as those experienced in ST positioning [20]. A review of previous approaches to IOP management during ST procedures is presented here and includes the usage of intraoperative strategies, including various IOP-lowering pharmacologic agents as well as modified Trendelenburg positioning. 

After a thorough literature review, the authors did not find any prior reports using oral acetazolamide in the perioperative management of IOP. The authors have found several other strategies in the literature regarding perioperative management of IOP spikes during ST, as outlined in Table 1. Three studies evaluated the use of dexmedetomidine in the intraoperative setting [10-12]. Dexmedetomidine, an alpha-2 agonist, decreases IOP by decreasing aqueous humor production. Joo et al. administered a loading dose of 0.1 µg/kg dexmedetomidine prior to induction of anesthesia [10] in addition to a continuous infusion of 0.5 µg/kg/h throughout the procedure, whereas the other two studies administered an infusion of 0.4 µg/kg/h dexmedetomidine [11, 12]. Each demonstrated a significant reduction in IOP during ST positioning, although these studies were conducted in patients without preexisting ocular disease. 

Lee et al. reported the use of an intraoperative 500 mg acetazolamide bolus followed by continuous infusion of 100 g of 20% mannitol in a POAG patient when their IOP reached 35 mmHg measured with a tonometer [13]. The bolus of acetazolamide decreased IOP significantly 30 minutes after administration, and the mannitol infusion led to maintenance of IOP below 21 mmHg (range: 17-20 mmHg) for the remainder of the procedure [13]. This reactive approach normalized IOP but required intraoperative monitoring and timely intervention. 

Two studies randomized patients to receive either a balanced salt solution or dorzolamide-timolol (D-T) drops following anesthesia induction [14, 15]. Dorzolamide is a carbonic anhydrase II inhibitor, and timolol is a topical beta-adrenergic receptor antagonist. Both drugs act to decrease the production of aqueous humor, thereby decreasing IOP. In the first study utilizing D-T, the drops were administered once the patient’s pressure reached 40 mmHg measured via tonometer, and the results showed that this measure prevented further increases in IOP [14]. The subsequent study administered either D-T drops after induction of anesthesia or a balanced salt solution and found that the D-T group had significantly reduced IOP measurements throughout the surgery [15]. 

Raz et al. distributed a group of patients into a modified Z Trendelenburg (ZT) position to mitigate ST-induced IOP increases [16]. ZT positioning is a variant of the traditional Trendelenburg position in which the patient is placed supine with the head and shoulders kept horizontal while the lower body is elevated and tilted downward. Compared to the regular ST group, the patients in the modified ZT group had a lower average IOP of 4.6 mmHg. Yoo et al. tested the effects of sevoflurane and propofol on IOP but found no statistically significant difference in IOP throughout the time under anesthesia [17]. 

In contrast to these intraoperative strategies, this case demonstrates the successful use of preoperative oral acetazolamide as a prophylactic measure. Acetazolamide is a reversible carbonic anhydrase inhibitor that, in the eye, decreases the production of aqueous humor and lowers the intraocular pressure. For oral acetazolamide, peak plasma concentration and maximum pharmacologic effect occur within two to four hours after administration. The duration of action of standard formulations is eight to 12 hours, while extended-release formulations, like those given in this case, exert effects for up to 18 to 24 hours. For intravenous acetazolamide, the onset of action is within minutes, with the maximum pharmacologic effect observed at 15 minutes to one hour after administration. IV administration of acetazolamide is preferred for rapid reduction of intraocular pressure in the acute setting. The maximum effect of oral acetazolamide within this two- to four-hour range nicely coincides with the average maximum IOP pressure spike due to ST positioning. In addition, the regimen was well-tolerated by our patient and associated with stable postoperative IOP and no evidence of glaucomatous progression on RNFL and visual field testing. 

## Conclusions

In this case, the authors present an oral prophylactic treatment strategy for a patient with glaucoma undergoing surgery in prolonged ST. In contrast to prior studies focusing on intraoperative interventions, this strategy offers a proactive, cost-effective, simple approach to perioperative IOP control. Preoperative oral acetazolamide avoids the need for intraoperative IOP monitoring and care coordination and may maintain IOP stability throughout the perioperative period. Despite increasing recognition of ST-induced IOP risks, no standardized guidelines currently exist for perioperative IOP management in glaucoma patients. These findings highlight the potential utility of preoperative systemic regimens to lower IOP and warrant further prospective studies to evaluate the efficacy, safety, and generalizability of this approach to inform evidence-based perioperative IOP management guidelines in glaucoma patients. 
